# Incidence of neural tube defects and their risk factors within a cohort of Moroccan newborn infants

**DOI:** 10.1186/s12887-021-02584-5

**Published:** 2021-03-15

**Authors:** Khenata Forci, El Arbi Bouaiti, Mohamed Hassan Alami, Asmaa Mdaghri Alaoui, Amal Thimou Izgua

**Affiliations:** 1 Congenital Defects Research Team, Faculty of Medicine and Pharmacy, University Mohamed V Rabat, P.B: 174 Downtown Rabat, Rabat, Morocco; 2 Laboratory of Biostatistics, Clinical & Epidemiological Research, Faculty of Medicine and Pharmacy of Rabat, University Mohamed V Rabat, Rabat, Morocco; 3 “Les Orangers” Maternity and Reproductive Health Hospital of Rabat, CHU IBN SINA, Rabat, Morocco; 4 Dysmorphology and Congenital Anomalies Unit, Pediatric Department 2, HER, CHU IBN SINA, Rabat, Morocco; 5 Center for consultations and external explorations, HER, CHU IBN SINA, Rabat, Morocco

**Keywords:** Incidence, Morocco, Neural tube defects, Prevention, Risk factors

## Abstract

**Background:**

Neural tube defects (NTDs) are a group of birth defects that result from a partial or complete failure of the neural tube to close during embryogenesis. Their prevalence varies between 0.5 to 2 per 1000 births in countries without folic acid supplementation. The aim of our study is to assess the NTDs incidence and describe the risk factors within Moroccan newborn infants.

**Method:**

This is a descriptive study over a period of 5 and a half years including all births at “Les Orangers” Maternity and Reproductive Health Hospital of Rabat with notification of NTD cases, whether isolated or combined with other anomalies. Data were reported on pre-established sheets and on the teratovigilance registry. Statistical analysis was performed with SPSS version 18 statistical software.

**Results:**

During the study period, 43,923 births were recorded including 44 cases of neural tube defects, an incidence rate of 1 per 1000 births, with a female predominance; sex ratio = 0.8. These defects included anencephaly (50%), spina bifida (38.6%) and encephalocele (11.4%). The risk factors detected during this study include consanguinity (34%), consumption of fenugreek or other plants (36%), diabetes (4.5%) and medication (2.2%). A family history of malformation was reported in 6.8% of cases and among siblings in 4.5% of cases. The average maternal age was 30.38 ± 6.88 and the average gestational age was 36.80 ± 5.11. A quarter of mothers did not benefit from any medical monitoring during pregnancy while 59% did not take folic acid supplementation during the first trimester of pregnancy and none of them took B9 vitamin during the periconceptional period. The antenatal diagnosis was performed in 63% of cases. The mortality rate was 3.8 per 10,000 and 16% of cases evolved positively.

**Conclusion:**

NTDs require high intensity and multidisciplinary care which stresses the importance, in our context, of strengthening and optimizing acid folic supplementation strategies during the periconceptional period.

## Introduction

Neural tube defects (NTDs) are congenital malformations that appear at the embryonic development stage, between the 23rd and 27th day of embryonic life. They vary from a simple spina bifida to a complete anencephalomyelia. Structural malformations at the development level can affect the thoracic, lumbar or sacral (spina bifida) spines as well as the skull (anencephaly or encephalocele), while secondary joint-anomalies (rupture or deformation) can affect the lower limbs, the intestines, the bladder, the cerebellum and the cortex or the brain ventricles (in the case of myelomeningocele). Worldwide, NTDs affect 323,904 infants [1] and cause 88,000 deaths every year in addition to leaving 8.6 million people with disabilities [2, 3], and they are the cause behind 29% of neonatal deaths in low-income countries [4]. The prevalence of these malformations ranges from 0.5 to 2 per 1000 births in countries without folic acid supplementation [5]. The etiologies of these anomalies are complex and include several genetic as well as environmental factors. Our study aims are to assess the NTDs’ incidence and describe the risk factors within Moroccan newborn infants.

## Method

### Study design

This is a prospective study, based on the notification of all cases presenting an anomaly of NTD isolated or associated with other anomalies within the Maternity and Reproductive Health Hospital “Les Orangers” in Rabat from January 1st, 2011 as of June 30th, 2016; it’s a level III-reference maternity which receives pregnant women from all over Morocco and performs 8000 deliveries per year. Normal and high-risk pregnancies are managed and monitored within the maternity following standardized protocols including antenatal checkups and obstetrical ultrasounds. Within this study, we have included all women whose fetus or newborn showed an isolated or combined neural tube defect, whether diagnosed through a prenatal ultrasound or the systematic clinical exam at birth, regardless of the pregnancy term or outcome.

### Data collection

Once a case has been confirmed, the doctor approaches the parents to explain the baby’s situation and provide a prognosis. He also explains the method and objectives of our study in order to obtain an informed parents’ consent. A majority of the interviewed women are illiterate and, for cultural reasons, they have provided a verbal (i.e. without signature) consent to participate in the study. Each couple has provided his consent after receiving the necessary explanations from a three-person team of medical personnel. All collected data; anamnesis, socioeconomic status, consanguinity, history of malformations within the family or siblings, exposition to teratogens, mother’s serology, pregnancy characteristics in addition to the newborn’s characteristics were reported on pre-established sheets and on the teratovigilance registry. The evaluation of the prognosis of these newborns was based on monitoring the short-term progress (6 months). An outcome presented in the evolution indicates the survival rate at 6 months of cases whether they are operated on or not yet.

### Definitions

Neural tube defects include: [6].

- Exencephaly / anencephaly: is an anterior neuropore closure anomaly with the neural tube in a state of neural tube, with an extracranial protrusion of the brain. The latter will suffer from aggression and decay due to the contact with amniotic fluid to cause anencephaly.
- Myelomeningocele / spina bifida: a more or less extensive cord closure abnormality, causing the posterior arches of the thoracic, lumbar or sacral spine to open.
- Encephalocele / meningocele: is a developmental defect of the mesoblast leading to an opening of the skull with herniation of the meninges. It can be occipital, parietal or fronto-ethmoidal.

### Statistical analysis

The statistical analysis for this descriptive epidemiological study was performed with SPSS version 18 statistical software. Quantitative variables are expressed in averages +/− standard deviation and qualitative variables in numbers and percentages.

## Results

**Fig. 1:**
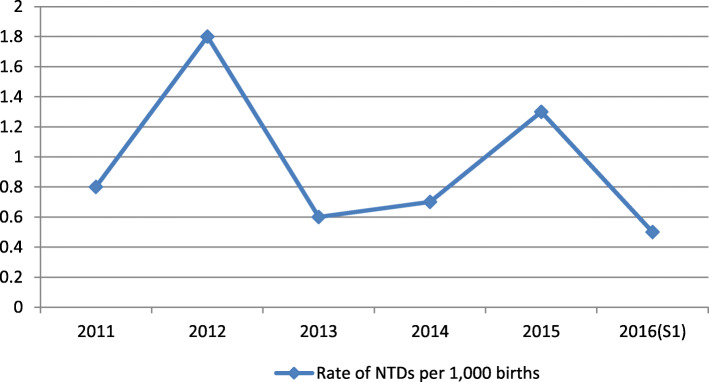
Yearly incidence rates of NTDs at “Les Orangers” Maternity and Reproductive Health Hospital of Rabat, Morocco. S1 = first semester

During the study period from January 1st, 2011 to June 30th, 2016, 43,923 births were recorded including 44 cases of neural tube defects; an incidence rate of 1 per 1000 births, this incidence rate varied throughout the years with 2 peaks. A first peak was recorded in 2012 with an estimated incidence rate of 1.8 per 1000 births (the highest rate recorded during the study period) and a second peak in 2015 with an incidence rate of 1.3 per 1000 births. The reported incidence rate for the 6-month period in 2016 was 0.5 (Fig. 1).

**Fig. 2:**
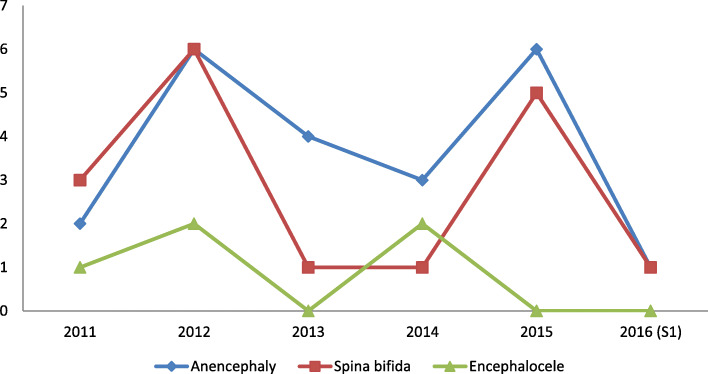
Yearly incidence rates of different types of NTDs at “Les Orangers” Maternity and Reproductive Health Hospital of Rabat, Morocco. S1 = first semester

The incidence rates for the various types of NTDs highlights 2 peaks of anencephaly and Spina Bifida; the first one in 2012 with an incidence rate of 6 per 1000 births and the second one in 2015 with an estimated rate of 5–6 per 1000 births. Encephaloceles’ incidence peaked twice in 2012 and 2014 with an estimated rate of 2 per 1000 births. (Fig. 2).

Half of reported defects were anencephaly (50%), followed by spina bifida (38.6%) and encephalocele (11.4%). Thus, the incidence rates of anencephaly, spina bifida and encephalocele were respectively 5, 3.8 and 1.1 per 10,000 births with a female predominance especially for anencephaly (sex ratio = 0.75) and encephalocele (0.33) while we noted a male predominance for spina bifida (1.33). Certain risk factors for NTDs described in the literature were detected in our series (Table 1), such as consanguinity wich was reported in 34% of cases, consumption of fenugreek and other plants in 36% of cases, diabetes in 4.5% and medication in 2.2%. A history of malformation was reported in the family for 6.8% of cases and among siblings for 4.5%. Half of women came from a low socioeconomic status, especially in anencephaly cases. The average maternal age was 30.38 ± 6.88 and the average gestational age was 36.80 ± 5.11. A quarter of mothers did not benefit from medical monitoring during pregnancy while 59% did not take folic acid supplementation during the first trimester of pregnancy and none of them took B9 vitamin during the periconceptional period. The antenatal diagnosis was performed in 63% of cases, with only 4 cases (9.1%) choosing a therapeutic interruption of pregnancy. We resorted to a caesarean section in 7 cases of NTDs.

**Table 1:** Social, demographic and obstetrical characteristics of deformed newborns’ mothers and main risk factors at “Les Orangers” Maternity and Reproductive Health Hospital of Rabat, Morocco

	44 cases of NTDs	Anencephaly 22 (50%)	Spina Bifida 17 (38.6%)	Encephalocele 5 (11.4%)
**Maternel age**				
< 35 years	33 (75)	16 (72.7)	14 (82.4)	3 (60)
≥ 35 years	11 (25)	6 (27.3)	3 (17.6)	2 (40)
**Socio-economic level**				
Low	26 (59)	12 (54.5)	11 (64.7)	3 (60)
Middle	15 (34)	8 (36.4)	5 (29.4)	2 (40)
High	3 (6.8)	2 (9.1)	1 (5.9)	0
**Consanguinity**	15 (34)	7 (31.8)	5 (29.4)	3 (60)
First degree	12 (27)	5 (22.7)	5 (29.4)	2 (40)
Second degree	3 (6.8)	2 (9)	0	1 (20)
Third degree	0	0	0	0
**Medical history**				
Diabetes	1 (2.27)	1 (4.5)	0	0
Gestational Diabetes	1 (2.27)	0	1 (5.9)	0
Malformation history in siblings	2 (4.5)	1 (4.5)	0	1 (20)
Malformation history in family	3 (6.8)	2 (9.1)	0	1 (20)
**Fenugreek consumption**	10 (22.7)	8 (36.4)	2 (11.8)	0
**Other plants consumption**	6 (13.6)	5 (22.7)	1 (5.9)	0
**Medication**				
Paracetamol	1 (2.27)	0	1 (5.9)	0
**Folic acid supplementation**				
In preconception	0	0	0	0
During the first trimester	11 (25)	5 (22.7)	5 (29)	1 (20)
**Pregnancy follow-up**	33 (75)	15 (68.2)	14 (82.4)	4 (80)
**Gestational age**				
≤ 21WA	3 (6.8)	0	2 (11.8)	1 (20)
22-36WA	9 (20.4)	6 (27.3)	2 (11.8)	1 (20)
≥ 37WA	32 (72.7)	16 (72.7)	13 (76.4)	3 (60)
**Prenatal diagnosis**	28 (63)	17 (77.3)	8 (47.1)	3 (60)
**Mode of delivery**				
Normal	36 (81.8)	20 (90.9)	11 (68.8)	5 (100)
Caesarean section	7 (16)	2 (9.1)	5 (31.2)	0
**TIP**	4 (9.1)	2 (9.1)	1 (5.9)	1 (20)

*NTDs* neural tube defects, *%* percentage, *WA* week of amenorrhea, *TIP* therapeutic interruption of pregnancy

The NTDs were combined with other malformations in 45.4% of cases (Table 2). Regarding the evolution of NTD cases, the 6-month survival rate is 16% represented by the 7 cases of spina bifida (Table 3).

**Table 2:** Distribution of NTD cases between isolated and combined with other congenital malformations at “Les Orangers” Maternity and Reproductive Health Hospital of Rabat, Morocco

NTDs	Total Number of cases		Isolated NTDs		Associated NTDs	
	Number	%	Number	%	Number	%
Anencephaly	22	50	18	81.8	4	18.2
Spina Bifida	17	38.6	4	23.5	13	76.5
Encephalocele	5	11.4	2	40	3	60

*NTDs* neural tube defects, *%* percentage

**Table 3:** Newborns’ characteristics and outcomes at “Les Orangers” Maternity and Reproductive Health Hospital of Rabat, Morocco

	NTDs (44) N (%)	Anencephaly 22 (50%)	Spina bifida 17 (38.6%)	Encephalocele 5 (11.4%)
**Newborn sex**				
Feminine	22 (50)	13 (59.1)	6 (35.3)	3 (60)
Male	19 (43.2)	9 (40.9)	9 (53)	1 (20)
Ambiguity	3 (6.8)	0	2 (11.7)	1 (20)
**Gestational age**				
≤ 21WA	3 (6.8)	0	2 (11.8)	1 (20)
22-36WA	9 (20.4)	6 (27.3)	2 (11.8)	1 (20)
≥ 37WA	32 (72.7)	16 (72.7)	13 (76.4)	3 (60)
**Mode of delivery**				
Normal	36 (81.8)	20 (90.9)	11 (68.8)	5 (100)
Cesarean section	7 (16)	2 (9.1)	5 (31.2)	0
**Birth weight**				
< 2500 g	26 (59)	15 (68.2)	7 (41.2)	4 (80)
[2500; 4000 g ]	15 (34)	6 (27.3)	8 (47.1)	1 (20)
≥ 4000 g	1 (2.3)	0	1 (5.9)	0
Unknown	2 (4.5)	1 (4.5)	1 (5.9)	0
**Apgar score**				
0	17 (38.6)	13 (59)	2 (11.8)	2 (40)
[1–3] to 1 min	12 (27.3)	6 (27.3)	4 (23.5)	2 (40)
[3–7] to 1 min	6 (13.6)	3 (13.6)	3 (17.6)	0
10 to 5 min	9 (20.5)	0	8 (47)	1 (20)
**Non malformative neonatal pathology**				
Respiratory distress	3 (6.8)	0	3 (17.6)	0
Neonatal suffering	3 (6.8)	1 (4.5)	2 (11.8)	0
Intrauterine growth retardation	11 (25)	4 (18.2)	5 (29.4)	2 (40)
Prematurity	5 (11.3)	1 (4.5)	3 (17.6)	1 (20)
**Newborn transfer**	3 (6.8)	0	3 (18.8)	0
**Transfer service**				
Neonatal intensive care unit	1 (2.3)	0	1 (33.3)	0
Pediatric ward	2 (4.5)		2 (66.7)	
**Evolution at 6 months**				
Living	7 (16)	0	7 (41.2)	0
Deceased	17 (38.6)	9 (40.9)	6 (35.3)	2 (40)
Fetal death in utero	17 (38.6)	13 (59.1)	2 (11.8)	2 (40)
Lost to follow-up	3 (6.8)	0	2 (11.8)	1 (20)

*NTDs* neural tube defects, *%* percentage, *N* number, *WA* week of amenorrhea, *g* grams, *min* minute

## Discussion

In our study, 44 cases of neural tube defect were reported, representing a prevalence rate of 10 per 10,000 births. This is a lower rate than those reported by previous Moroccan studies. Thus (Table 4) presents a comparative study of the rate of NTDs in different countries in the world and Morocco. The decrease in NTDs in Morocco is due to the implementation since 2008 by the health ministry, of a supplementation strategy to provide every pregnant women with 400 μg of folic acid and a daily dose of 5 mg to every women under antiepileptic treatment (sodium valproate, carbamazepine) or with a history of NTDs (had given birth to a baby with NTD, or NTD cases within her family) 2 months prior to conception and during the first trimester of pregnancy [30, 31].

**Table 4:** Comparative table of NTDs’ prevalence rates per 10,000 births in different countries in the world and Morocco

Country and study period	NTD’s prevalence rates per 10,000 births	Specific prevalence rates per 10,000 births			Incidence rates per 1000 births
		Anencephaly	Spina Bifida	Encephalocele	
**In Africa**					
Algeria					
- 2004–2006 [7]	75.3	32.2	42.8	0.3	7.5 [8]
- 2012–2013 [9]	15.8				1.58
Tunisia [10]					2.2
Libya 1995 [11]		7.4	0.6		
Congo [12] 1993–2001	10.2	1.1	6.8	2.3	1
Ghana [13] 1991–1992		8.4	3.1		
Nigeria [14] 1980–2003		1.6	3.7		
Morocco					
- 2008–2011 [15]	21.78–12.1				
- 2012–2015 [16]	10.5				
- 2011–2016	10	5	3.8	1.1	1
**In Europe** 2011–2017 [17]	10.08	4.05	4.91	1.12	1
France					
(Rhône Alps)	12.51	5.24	6.00	1.27	1.2
(Auvergne)	11.73	4.82	5.48	1.43	1.17
Belgium (Antwerp)	5.74	1.70	3.64	0.40	0.57
Germany (Mainz)	13.11	4.63	6.94	1.54	1.3
Sweden	7.12	2.64	3.76	0.72	0.7
Denmark (Odense)	14.17	4.72	7.99	1.45	1.4
Italy (Emilia Romagna)	5.36	1.74	2.70	0.93	0.5
Spain (Basque country)	11.04	6.20	4.16	0.69	1.1
England and Wales					
- 2000 [18]	16.3				1.6
- 2004 [19]	12.8				1.3
**In American continent**					
Costa Rica [20]					
- 1987	9.8				0.98
- 2012	4.8				0.48
USA [21, 22]					
- 1999–2007	5.25	1.3	3.17	0.78	0.52
- 1995–2002					
Hispanics		4.2	2.8		
Non-Hispanic whites		3.4	2		
Non-Hispanic blacks		2.9	1.8		
Mexico 2008–2013 [23]	3.2	1.52	1.39	0.28	0.32
Canada [24]					
- 1996	7.6	1.1	5.5	1.1	0.76
- 2004–2007	4	0.8	2.7	0.6	0.4
**In Asia**					
India 2011–2012 [25]	17.9	1.6	14	2.3	1.8
China 2007–2009 [26]	11.3	6.3	3.6	1.4	1.1
Japan 2011 [27]	6.23	0.32	5.59	0.32	0.6
Turkey 2003–2004 [28]	35.8	13.9	19.6	2.3	3.58
Iran 1998–2005 [29]	25.4	11.4	12.7	1.3	2.5

The disparity in NTD rates in Africa is due to several factors; distinctive characteristics in terms of geography, genetics, culture and nutrition in addition to differences in health policies between countries (i.e. whether or not a folic acid supplementation strategy has been implemented). The reported rates are also sensitive to the type, duration, sample size and methodology of the study.

In developed countries, this decrease in the rate of NTDs is due to the mandatory folic acid supplementation and fortification especially during the preconception period, early screening through prenatal diagnosis and therapeutic interruption of pregnancy.

In our study, we observed a female predominance especially in cases of anencephaly (sex ratio = 0.75) which was similarly described by Houcher et al. [7]. On the other hand, we observe a male predominance in cases of spina bifida (1.33) which was also described by Rai et al. [32]. NTDs can have multifactorial causes; genetic or environmental, or resulting from an interaction between genetic and environmental factors. Recent studies have reached the conclusion that the presence of C677T coding gene 5.10- Methylene Tetrahydrofolate Reductase, an enzyme which affects folate synthesis, increases the risks of spina bifida and anencephaly in the fetus [33]. Other risk factors may be associated, notably diabetes and obesity [34, 35]. Some drugs, such as valproic acid, carbamazepine, fumonisin, trimethoprim and warfarine anticoagulant, can also cause NTDs. Valproic acid, for instance, is responsible for 2 to 3% of spina bifida cases [36]. Other risk factors have been identified such as hyperthermia, zinc deficiency and some cleaning products [37]. However, folate deficiency remains the most widely recognized risk factor and can be prevented with very simple measures. Certain risk factors for NTDs described in the literature were detected in our series, such as consanguinity (34%), consumption of fenugreek and other plants (36%), diabetes (4.5%) and medication (2.2%). A case-control study was conducted between 2008 and 2011 in Morocco which reported that the association of low socio-economic status was statistically significant in the occurrence of NTDs [15], this is due to malnutrition especially the deficiency of folic acid and insufficient monitoring of pregnancy, in our study 59% of women belong to a low socio-economic population. Several studies have reported the role of consanguinity in the occurrence of congenital malformations. What is more, these malformations are twice as likely to occur in the case of first cousins [38]. According to Talbi et al., Morocco has one of the highest consanguinity percentage at 22.79% and a consanguinity coefficient of 0.0088 [39]. In our study, consanguinity was notified in different types of NTDs with an estimated rate of 34% and even 80% in first degree cases. This is a higher rate than the one observed by Bourouba et al. in Algeria where the consanguinity rate was 30% [9], and by Golalipour et al. in Iran who reported a rate of 28% [29]. The consumption of fenugreek and other plants was primarily related to cases of anencephaly and spina bifida. Fenugreek (*Trigonella foenum-graecum*) is a medicinal and seasoning plant widely used in Moroccan as well as Mediterranean and Asian cuisine. In Morocco, this plant is consumed to treat several diseases and its seeds are used for pregnancy-related ailments such as vomiting as well as to stimulate appetite [40]. In their analysis of studies on the clinical association between fenugreek consumption and the occurrence of NTDs, A. Es Seddiki et al. reached the conclusion that this plant represents a major risk factor and had detrimental fetotoxic and teratogenic effects on the fetus [41].

In our series, we have identified a history of malformations within siblings (4.5%) and within the family (6.8%). This recurrence risk has been described in literature with rates ranging from 2 to 5% [42]. However, several studies have concluded that a folic acid supplementation during the periconceptional period reduces this recurrence risk by 40 to 80% [4, 43–48]. Other studies proved the efficiency of folic acid supplementation in reducing NTD rates when taken during the periconceptional period and the first trimester [49–51]. However, none of the women in our study received a folic acid supplementation during the periconceptional period and as much as 60% of them did not receive it during the first trimester of pregnancy.

The NTDs’ antenatal diagnosis through obstetrical ultrasound allows us to identify the type of defect and any associated malformations. Thus, it enables a therapeutic decision and an informed discussion with the couple regarding options for in utero repair or a therapeutic interruption of pregnancy. In their study, Matuszewski et al. have observed a strong correlation between antenatal ultrasound data and postnatal examination of spinal dysraphism [52]. About two thirds (63%) of NTD cases in our study were diagnosed antenatally with only 4 cases (9.1%) who underwent a pregnancy termination procedure. This low rate is due to several factors, especially the couples’ religious and sociocultural beliefs that reject resorting to pregnancy termination in case of fetal anomalies.

Furthermore, the rate of associated malformations varies, depending on the study, between 18 and 46% for anencephaly, 12 and 63% for spina bifida and exceeds 80% for encephalocele [53]. Matuszewski et al. and Toru et al. reported a rate of NTD-associated malformations at 32.7 and 32% respectively [52, 54]. In our study, about half of NTD cases presented multiple malformations; 13 cases of spina bifida were associated with other types of malformations which represents 76.5% of cases. This is higher than the rate of 33% reported by De Vigan et al. [53]. Among our cases of spina bifida, 7 cases or 53.8% were associated with at least one hydrocephalus which is in line with the figures reported by Alatise et al. and Kumar R. et al. at respectively 53.8 and 58.8% [55, 56]. Studies in developed countries have reported higher rate between 78 and 86% [57] or between 85 and 90% [58].

Cases of fetal death in utero (FDIU) represented 38.6% while 17 cases (38.6%) died before or during the medical care. Our mortality rate of 3.8 per 10,000 total births with a fatality rate of 38% is significantly higher than the rate of 10% reported in literature [52]. This is mainly due to severe NTD cases especially of anencephaly and multiple malformations in addition to the challenges of providing adequate medical care to NTD cases in our context.

### Study limitations

This study covers one medical center. In order to notify all abnormalities including functional abnormalities, it is necessary to make a long-term follow-up, which was not the case in the present study. Therefore, we need to consider a wider study which will allow us to assess the incidence rate at the national level, and to look into the implementation of primary preventive measures in order to assess the efficiency of folic acid supplementation and fortification during the preconceptional and pregnancy periods.

## Conclusion

Through our study of NTDs’ incidence at “Les Orangers” Maternity and Reproductive Health Hospital of Rabat, we observed a rate of 1 per 1000 births. Among the risk factors for NTDs described in the literature, consanguinity and the consumption of fenugreek are frequent in our context and require preventive actions. In order to reduce the rate of NTDs in our context, the prevention strategy implemented by the Ministry of Health should be further strengthened by enforcing an early folic acid supplementation during the preconception period, monitoring pregnancies, increasing women’s awareness of the importance of consuming folate-rich food and banning any consumption of fenugreek during the first trimester of pregnancy. Finally, the implementation of a national program for prenatal NTD screening will allow us to provide more adequate medical care.

## Data Availability

The datasets used and /or analysed during the current study are available from the corresponding author on reasonable request.

## Abbreviations

NTDs: Neural tube defects
TIP: Therapeutic interruption of pregnancy
FDIU: Fetal death in utero
USA: United States of America
